# Role of a Mixture of Polyphenol Compounds Released after Blueberry Fermentation in Chemoprevention of Mammary Carcinoma: In Vivo Involvement of miR-145

**DOI:** 10.3390/ijms24043677

**Published:** 2023-02-12

**Authors:** Jean-François Mallet, Roghayeh Shahbazi, Nawal Alsadi, Ammar Saleem, Agnes Sobiesiak, John Thor Arnason, Chantal Matar

**Affiliations:** 1 Department of Cellular and Molecular Medicine, Faculty of Medicine, University of Ottawa, 451 Smyth Road, Ottawa, ON K1H 8M5, Canada; 2 Laboratory for the Analysis of Natural and Synthetic Environmental Toxins, Department of Biology, University of Ottawa, 30 Marie Curie Private, Ottawa, ON K1N 6N5, Canada; 3 School of Nutrition Sciences, Faculty of Health Sciences, University of Ottawa, 451 Smyth Road, Ottawa, ON K1H 8M5, Canada

**Keywords:** polyphenols, cancer stem cells, metastasis, microRNAs, FOXO1, N-ras

## Abstract

Epigenetic mechanisms such as microRNA (miRNA) deregulation seem to exert a central role in breast cancer initiation and progression. Therefore, targeting epigenetics deregulation may be an effective strategy for preventing and halting carcinogenesis. Studies have revealed the significant role of naturally occurring polyphenolic compounds derived from fermented blueberry fruits in cancer chemoprevention by modulation of cancer stem cell development through the epigenetic mechanism and regulation of cellular signaling pathways. In this study, we first investigated the phytochemical changes during the blueberry fermentation process. Fermentation favored the release of oligomers and bioactive compounds such as protocatechuic acid (PCA), gallic acid, and catechol. Next, we investigated the chemopreventive potentials of a polyphenolic mixture containing PCA, gallic acid, and catechin found in fermented blueberry juice in a breast cancer model by measuring miRNA expression and the signaling pathways involved in breast cancer stemness and invasion. To this end, 4T1 and MDA-MB-231 cell lines were treated with different doses of the polyphenolic mixture for 24 h. Additionally, female Balb/c mice were fed with this mixture for five weeks; two weeks before and three weeks after receiving 4T1 cells. Mammosphere formation was assayed in both cell lines and the single-cell suspension obtained from the tumor. Lung metastases were counted by isolating 6-thioguanine-resistant cells present in the lungs. In addition, we conducted RT-qPCR and Western blot analysis to validate the expression of targeted miRNAs and proteins, respectively. We found a significant reduction in mammosphere formation in both cell lines treated with the mixture and in tumoral primary cells isolated from mice treated with the polyphenolic compound. The number of colony-forming units of 4T1 cells in the lungs was significantly lower in the treatment group compared to the control group. miR-145 expression significantly increased in the tumor samples of mice treated with the polyphenolic mixture compared to the control group. Furthermore, a significant increase in FOXO1 levels was noted in both cell lines treated with the mixture. Overall, our results show that phenolic compounds found in fermented blueberry delay the formation of tumor-initiating cells in vitro and in vivo and reduce the spread of metastatic cells. The protective mechanisms seem to be related, at least partly, to the epigenetic modulation of mir-145 and its signaling pathways.

## 1. Introduction

Transformation of medicinal plant products by microbial fermentation to produce new nutraceuticals is a common practice in Asia and Europe [1]. In recent years, fermented plant products have become popular globally due to their unique sensory properties and health benefits [2]. Fermented foods are a rich source of probiotics, prebiotics, and polyphenols with known health-promoting properties that potentially work by modulating gut microbiota and the immune system [2,3,4]. Microbial fermentation of plant products generates bioactive compounds by metabolizing fermentable macronutrients and improves the nutritional value, polyphenol levels, and antioxidant capacity [2,5]. Due to their high concentration of bioactive compounds, fermented products play a significant protective role against chronic inflammatory diseases such as type 2 diabetes, cancers, and cardiovascular disease [2,6].

Blueberries are a well-known source of phenolic compounds [7]. We previously showed that fermenting native North American blueberries (*Vaccinium corymbosum* (highbush blueberry) or *V. angustifolia* Aiton (lowbush blueberry)), using a novel bacterium, *Rouxiella badensis* subsp *acadiensis* (known as Canen SV-53) isolated from the blueberry skin microflora, significantly increases the amount of polyphenols present in the blueberry juice, raises its antioxidant potential [1,8], and improves its anti-inflammatory properties and health-promoting activities [9,10,11]. This fermented blueberry juice, known as polyphenol-enriched blueberry preparation (PEBP), decreases the formation of cancer stem cells (CSCs) and notably suppressed the metastasis of breast cancer cells to the lungs in a mouse model of breast cancer [6].

Furthermore, we have shown that PEBP has potential chemopreventive properties through the epigenetic modulation of CSCs’ self-renewal pathways [6,12,13]. CSCs are a small subset of neoplastic cells which may contribute to tumor growth, maintenance, and recurrence [13]. Moreover, we have obtained evidence that carcinogenesis in breast cancer was regulated by epigenetic-specific changes that involved microRNAs (miRNAs) [14]. Epigenetic changes mediated by miRNAs contribute to CSCs characteristics, including self-renewal ability, mammospheres formation, and chemoresistance by modification-specific signaling involved in their survival and proliferation [15]. Our previous research found an upregulation of tumor suppressor miR-145 expression and a significant downregulation of oncogenic miR-210 expression in 4T1 and MDA-MB-231 breast cancer cell lines treated with PEBP [12]. We also found an increase in the Forkhead box O1 (FOXO1) level and a decrease in the N-ras level in cells exposed to PEBP [12]. However, no detailed studies of the phytochemical changes that occurred after fermentation have been published thus far.

In this research, our primary objective was to identify quantitative phytochemical changes during the fermentation process. As a first step, we employed untargeted metabolomics using ultra-performance liquid chromatography-quadrupole time-of-flight mass spectrometry (UPLC-MS-QTOF) analysis to identify compounds present in fermented and unfermented juice. Discriminant analysis was used to identify the significant phytochemical markers present in the fermented juice compared to non-fermented juice. Next, targeted analysis allowed us to measure the amounts of changes caused by the fermentation. Using our library of compounds and a modified validated methods we used before for blueberry products, we applied targeted analysis to better characterize the full range of changes in the fermented product. Our results showed that fermentation favored the release of small oligomeric and bioactive compounds such as protocatechuic acid (PCA), gallic acid, and catechol.

Our secondary objective was to investigate the possible mechanism of action of a polyphenol mixture (PCA mix) containing PCA, gallic acid, and catechin that was released after blueberry juice fermentation against the development of breast cancer. To this end, we measured the effect of the mixture on the expression of the targeted miRNAs and proteins involved in breast cancer cell proliferation, CSCs’ self-renewal, and tumor formation using a 4T1cell-induced breast cancer model. In this paper, we report that some polyphenol compounds found in fermented blueberry juice decreased the formation of cancer stem cells, delayed the development of mammary carcinoma tumors, and inhibited the metastasis of the highly metastasizing 4T1 cells to the lungs in animals receiving the polyphenolic compounds.

## 2. Results

### 2.1. UPLC-QTOF Analysis of Fermented and Non-Fermented Blueberry Juice

**Figure 1:**
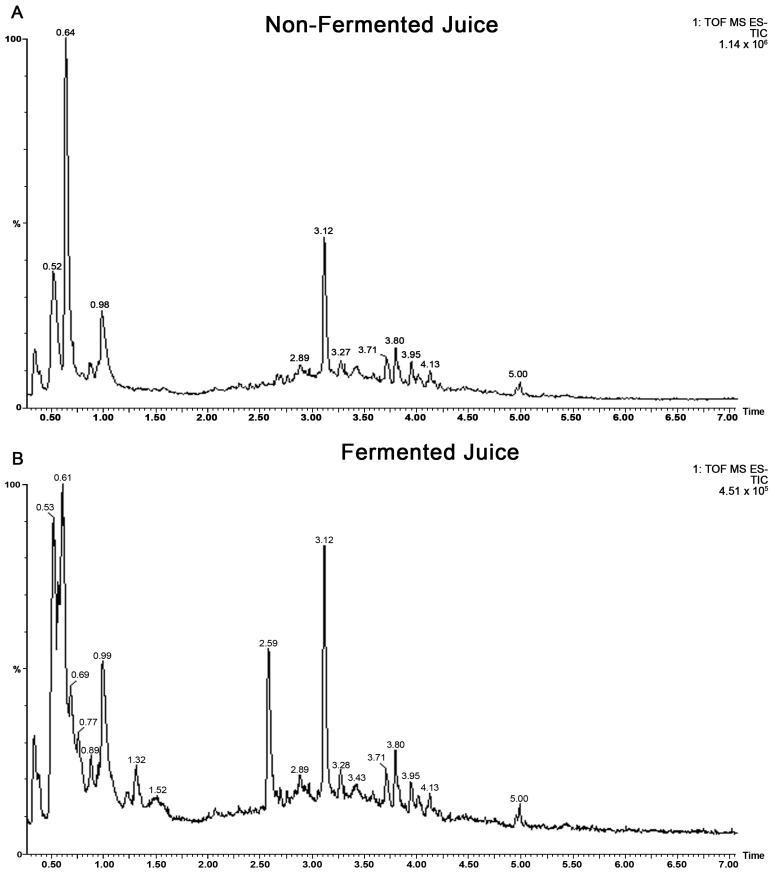
Total ion chromatograms (TOF ESI negative) of (**A**) non-fermented and (**B**) fermented blueberry juice by SV-53.

The total phenolic content was increased from 5.9 mM Gallic Acid Equivalent (GAE) to 30.7 mM GAE in fermented juice, confirming successful biotransformation. Untargeted metabolomics using UPLC MS QTOF were used to identify the compounds present in fermented and non-fermented juice. Gradient separation was developed using reversed-phase UPLC and the sub-two micron particle size stationary phase. By applying this high-resolution separation, the compounds were well separated within 18 min. Negative electrospray ionization was the best approach for phenolic acids and flavonoids, while positive ionization was preferable for anthocyanins using quadrupole time of flight mass spectrometry. Although the profiles of fermented and unfermented juice are similar (Figure 1A,B), qualitative changes in specific compounds were visible in the chromatograms. Once the analysis was completed using optimal conditions, the identification of individual metabolites was carried out.

### 2.2. Identification, Quantification, and Discriminant Analysis of Metabolites

**Figure 2:**
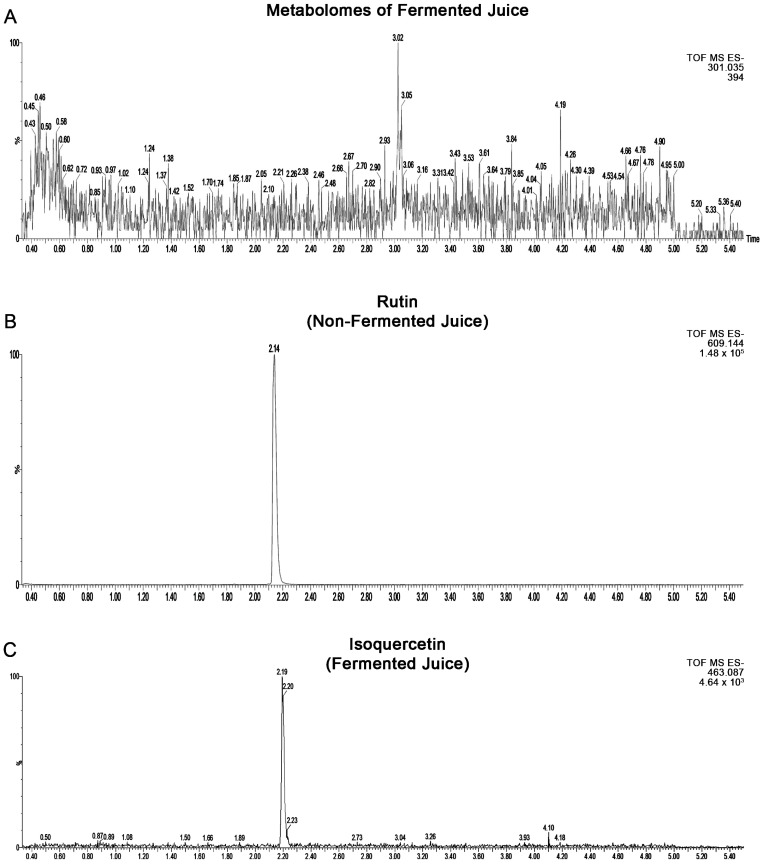
(**A**) Metabolomes detected in fermented blueberry juice by SV-53. Extracted ion spectrum of (**B**) rutin in non-fermented and (**C**) isoquercetin in fermented blueberry juice.

Figure 2A illustrates the metabolites detected in fermented blueberry juice. The use of discriminant analysis with metabolomics data is a means of identifying potentially bioactive compounds in fermented plant extracts and is complementary to bioassay-guided isolation [16,17]. Therefore, a search was conducted for at least 114 small molecules (mostly secondary metabolites), known in Vaccinium species, by UPLC-QTOF electrospray ionization (positive and negative modes). Of these compounds, confirmed identification was made for 22 compounds detectable in the study materials within 5 ppm mass accuracy and based on retention matching with authentic standards. These confirmed identified compounds mainly include phenolic acids, flavonoids, epicatechin, Myricetin-3-*O*-galactoside, Myricetin-3-*O*-glucoside, Quercetin-3-*O*-galactoside, Quercetin-3-*O*-glucoside, Quercetin-3-*O*-rhamnoside, Quercetin, anthocyanins, and procyanidins (Figure 2A). See Table 1 for more details about the compounds’ features. In addition to these compounds with confirmed identification, tentative identification of several compounds was made using spectral matches with online databases. These compounds included carbohydrate metabolites galactonic acid, glucuronic acid, 4-*O*-β-δ-glucopyranosyl-δ-glucose, a bacterial secondary metabolite, pramicidin, and gallotanin.

Furthermore, the metabolomes of fermented and non-fermented juice were subjected to discriminant analysis to identify key markers that differentiate the two samples. The most significant markers in the fermented juice were catechol, gallic acid, and gluconic acid. Catechol and gallic acid are visible in the large peaks in fermented juice in Figure 1B but absent in the non-fermented juice in Figure 1A.

We also observed a decrease in the level of rutin and a rise of its aglycone counterpart, quercetin. This suggest that rutin is a possible substrate for SV-53. (Figure 2B,C).

### 2.3. Effect of the Polyphenolic Mixture on Mammospheres Formation in 4T1 and MDA-MB-231 Cell Cultures

**Figure 3:**
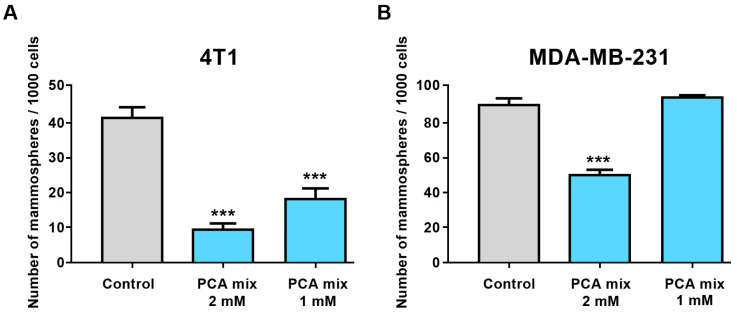
The number of mammospheres formation from (**A**) 4T1 and (**B**) MDA-MB-231 cell lines in a low attachment environment exposed to 1 or 2 mM gallic acid equivalent of the protocatechuic acid-based mixture (PCA Mix) for 4–7 days. One-way ANOVA followed by Dunnett’s post hoc test was used to compare groups. All values are means of three separate experiments ±SEM. *** *p* ≤ 0.001 vs. control.

First, we treated 4T1 and MDA-MB-231 cell lines with different concentrations of the polyphenolic mixture, ranging from 0.5 mM to 3 mM GAE to optimize the best doses for treating cells to conduct subsequent experiments. Cell viability was assessed by water-soluble tetrazolium salts (WST-1) and Lactate Dehydrogenase (LDH) assays (Roche, Laval, QC, Canada). We then selected 1- and 2-mM GAE concentrations of the polyphenolic mixture to perform our experiment. Treatment of 4T1 cells with 1- and 2-mM GAE of the polyphenolic mixture for 24 h significantly decreased the formation of mammospheres in this cell line (Figure 3A). However, only higher concentrations (2 mM GAE) significantly inhibited mammospheres formation in MDA-MB-231 cells (Figure 3B). 

### 2.4. Effect of the Polyphenolic Mixture on FOXO1 and N-ras Expressions in 4T1 and MDA-MB-231 Cell Lines

**Figure 4:**
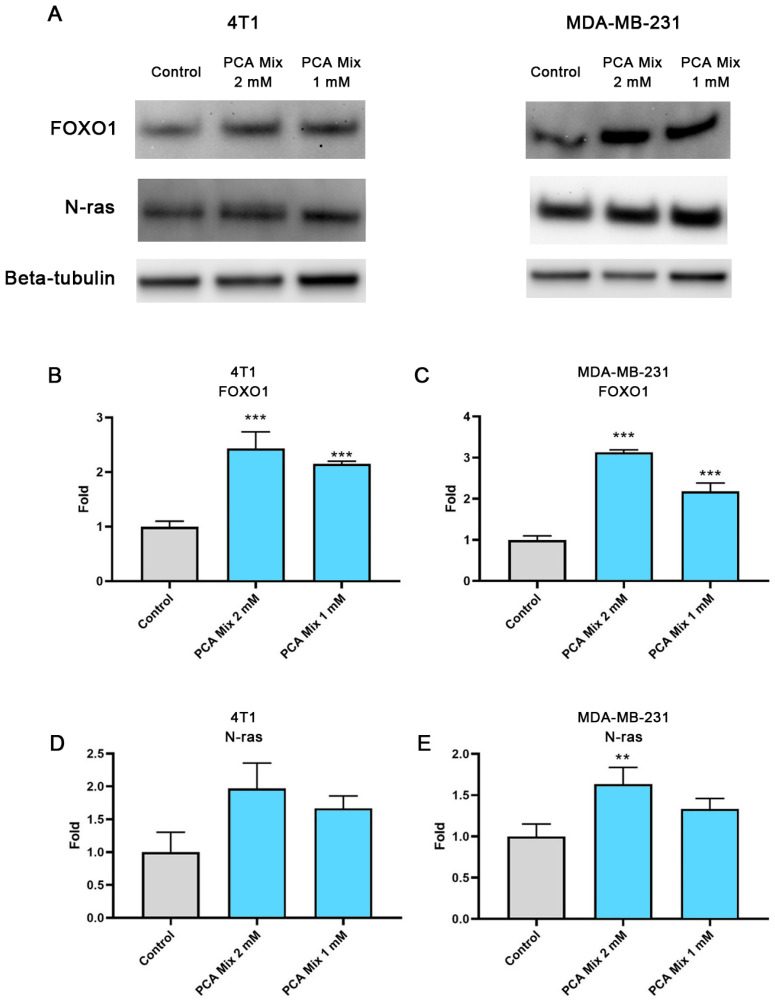
Relative expression of FOXO1 and N-ras in 4T1 and MDA-MB-231 cells exposed to 1 or 2 mM gallic acid equivalent (GAE) of a protocatechuic acid-based mixture (PCA mix) for 24 h. (**A**) A representative Western Blot. (**B,C**) Relative expression of FOXO1 in 4T1 and MDA-MB-231 cells, respectively, and (**D**,**E**) relative expression of N-ras in 4T1 and MDA-MB-231 cells, respectively. One-way ANOVA followed by Dunnett’s post hoc test was used to compare groups. All values are means of three separate experiments ±SEM. ** *p* ≤ 0.01 and *** *p* ≤ 0.001 vs. control.

4T1 and MDA-MB-231 cells were exposed to 1 mM and 2 mM GAE of the mixture for 24 h in order to examine the level of FOXO1 and N-ras expression in cell cultures. FOXO1 is a major tumor suppressor which controls cell proliferation. Dysregulation of FOXO1 is thought to contribute to the progression of a variety of cancers, including breast carcinoma [18]. Furthermore, FOXO1 might inhibit N-ras activation by regulating miRNA expression, mainly miR-145. N-ras overexpression has been linked with the formation and progression of breast cancer [12]. Treatment of 4T1 and MDA-MB-231 cells with the 1- and 2-mM GAE of the polyphenolic mixture significantly elevated the expression of FOXO1 in cells (*p* ≤ 0.001) (Figure 4A,B). We also observed a significant increase in N-ras levels in MDA-MB-231 cells treated with 2mM GAE of the polyphenolic mixture (*p* ≤ 0.01), while no change was observed in 4T1 cells (Figure 4C,D).

### 2.5. Effect of the Polyphenolic Mixture on miR-145 and miR-210-5p Expressions in Tumor Samples

**Figure 5:**
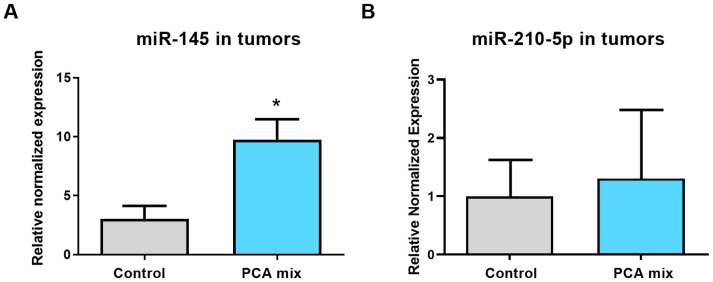
Relative expression of (**A**) miR-145 and (**B**) miR-210-5p in tumors from mice receiving either drinking water (control group) or a polyphenolic mixture (PCA mix) for five weeks. Independent *t*-test was performed to compare groups. All values are means of three separate experiments ±SEM (for a total of 12 animals in each group). N = 12 in each group. * *p* < 0.05 vs. control.

We previously conducted a microarray experiment to find the differentially expressed miRNAs in the 4T1 cell line exposed to PEBP for 24 h [12]. Our microarray analysis, followed by validation using qRT-PCR, revealed and confirmed the over-expression of the tumor suppressor miR-145 and under-expression of the oncogenic miR-210 in 4T1 cells [12]. Therefore, in the present study, we assayed the expression of miR-145 and miR-210-5p in 4T1-induced mammary tumors collected from mice treated with our polyphenolic mixture for a five-week period. Our result revealed a significant increase in miR-145 expression in the tumor samples of mice treated with the polyphenolic mixture compared to the control group (*p* < 0.05) (Figure 5A); however, no significant difference was observed in the expression level of miR-210-5p (Figure 5B).

### 2.6. Effect of the Polyphenolic Mixture on Spheroids Formation and Metastasis Ex Vivo

**Figure 6:**
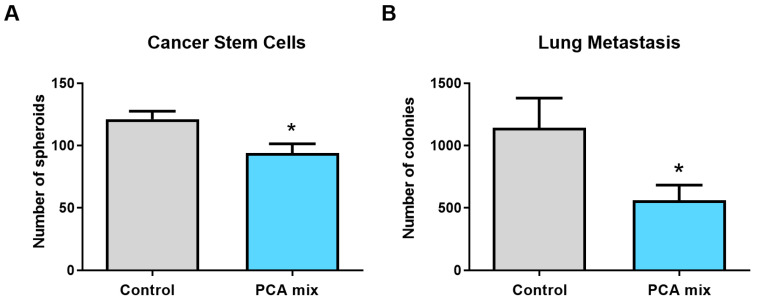
(**A**) The number of spheroids from cell isolates of 4T1 cell-induced tumors and (**B**) the number of colony-forming units of 4T1 cells present in the lungs of mice receiving either drinking water (control group) or a polyphenolic mixture (PCA mix) for five weeks. Independent *t*-test was performed to compare groups. All values are means of three separate experiments ±SEM. N = 12 in each group. * *p* < 0.05 vs. control.

Spheroids formation from tumoral primary cells was significantly reduced in tumors removed from animals fed with polyphenols (*p* < 0.05) (Figure 6A). Similarly, the number of colony-forming units of 4T1 cells present in the lungs of mice was significantly lower in the treatment group compared to the control group, indicating the reduction of the metastasis in the lungs of polyphenols-treated mice (*p* < 0.05) (Figure 6B).

## 3. Discussion

Naturally occurring compounds, mainly polyphenols, have gained immense attention because of their ability to target key inflammatory signaling pathways [6,12,19]. Numerous studies are currently focused on developing innovative phytochemical-based treatment options for the prevention and treatment of cancer [20]. We have provided evidence that fermented blueberry juice, referred to as PEBP, exhibits a potential chemopreventative role in cancer [6]. The molecular mechanisms underlying the pleiotropic activities of fermented products produced by SV-53 involve the regulation of global cell regulators at various levels of cell signaling, which are implicated in inflammatory response and immune homeostasis [6,12,13].

Herein, we first aimed to study phytochemical changes in blueberry juice following the fermentation process. The transformation by SV-53 leads to an increase in bioactive components, resulting in PEBP having four times more antioxidant activity than normal blueberry juice [1]. One hypothesis that underlines the higher beneficial effects of PEBP is related to tannin degradation, which converts large polyphenols to smaller oligomers. Small oligomers are known to be better absorbed, greatly affecting their bioavailability and consequently their physiological effects [21]. Small oligomers of polyphenols may then exert their activity as prebiotics or natural ligands for the toll-like receptors (TLRs) involved in immune regulation. In fact, there is a growing body of evidence to support the notion that some polyphenolic ingredients act as prebiotics. For example, quercetin has proven to positively influence microbiota [22]. Quercetin is an important flavonol with known anti-inflammatory activities. Interestingly, quercetin might exert its anti-inflammatory activity via the blockade of the TLR4-mediated signaling pathway [6,23]. In addition, quercetin has been found to increase anti-inflammatory miR-200b and miR-145 in pancreatic and ovarian cancer stem cells, respectively [24,25]. Along this line, the presence of isoquercetin in the fermented blueberry juice might indicate that SV-53 is able to hydrolyze the sugar moiety in rutin and thereby enrich it with bioactive phenolic acids such as PCA. This is one of many examples of how the fermentation of blueberries might yield bioactive compounds positively influencing ligands found on non-immune and immune cells, differentially influencing the miRNAs profile.

Blueberry polyphenols have been widely studied for their wide range of health benefits [2]. Although more than 8000 polyphenols have been discovered [26], research has focused on a specific class of flavonoids known for their beneficial effects, including quercetin, rutin, catechin, and PCA [27]. The protective effects of flavonoids are not only due to intact flavonoids, as their bioavailability in their native form is low, but also or exclusively due to other bioactive substances formed after microbial degradation by gut microbiota [21]. PCA, a metabolite of quercetin, has a remarkable antiatherogenic effect. PCA, as the gut microbiota metabolite of cyanidin-3-*O*-β-glucoside (Cy-3-G), exerts its antiatherogenic effect partially through miRNA-10b [28]. PCA was also shown to have an apoptotic effect on cancer cells [29].

In perfect alignment with these observations, we showed that our biofermentation process mimics a healthy colonic fermentation of flavonoids by colon microbiota. In fact, phytochemical studies using UPLC-QTOF analysis revealed a significant change in the fermented product compared to conventional juice. The biofermentation process led to the appearance of novel peaks of oligomeric phenols. We have also shown the release of gallic acid, catechol, chlorogenic acid, and PCA in fermented blueberry juice [30]. Additionally, we have demonstrated that our probiotic can transform rutin into its aglycone counterpart quercetin. Furthermore, the biofermentation of quercetin generated a wide range of metabolites, including p-hydroxyphenylacetic acid, PCA, 3-(4-hydroxyphenyl) propionic acid, p-hydroxybenzoic acid, and p-coumaric acid [31]. Notably, the main metabolite produced through the colonic fermentation of quercetin is PCA [31]. 

Next, we examined the preventative effect of a PCA-based polyphenolic mixture, consisting of protocatechuic acid, gallic acid, and catechin, which are the main polyphenolic compounds found in fermented blueberry juice produced by the novel probiotic bacterium SV-53. Our main goal was to study the inhibitory effect of this mixture on CSC formation and metastasis through the regulation of specific signaling pathways and miRNA expression, both in vitro and in vivo. 

CSCs are the key drivers of cancer and play a role in relapse, resistance to anticancer therapies, and tumor recurrence [32]. CSCs derived from breast cancer cells with CD44+/CD24 low/− phenotype have the ability of heterogeneous differentiation, initiating diverse tumors and forming mammospheres [33,34,35,36]. The mammosphere formation assay has been used as a useful method for studying stem cell-like characteristics in breast cancer cell cultures [35]. Polyphenols such as resveratrol and curcumin have been found to exhibit cytotoxic effects on CSCs, eliminate CSC populations from tumors, inhibit the formation of mammospheres, and thus prevent tumor formation [37]. Accordingly, we have previously demonstrated that PEBP delays the formation of cancerous stem cells in different types of cell cultures and in vivo through modulation of IL-6/STAT3, as well as the extracellular regulated kinase (ERK) and p38 in mitogen-activated protein kinase (MAPK) signaling pathways [6]. The STAT3 and MAPK pathways play a crucial role in CSCs growth and metastatic characteristics [6]. In accordance with our previous results, we found that our polyphenolic mixture prevented mammosphere formation in vitro in 4T1 and MDA-MB-231 cell lines, and ex vivo in the cells isolated from breast tumors.

Epigenetic mechanisms, such as DNA methylation, histone modifications, and miRNAs contribute to the development of CSCs [36]. miRNAs can play either inhibitory or stimulatory roles in CSCs development [15]. For instance, miR-145, miR-200c, miR-494, and miR-34 have been shown to inhibit CSCs, while miR-19, miR-501-5p, miR-21, and miR-221/222 promote CSC development [15]. We have previously reported epigenetic-specific changes in CSCs that involve miRNAs [12,13]. We identified several differentially expressed clusters of the miRNAs involved in maintaining the inflammatory microenvironment and are associated with various clinical-pathological characteristics of breast cancer, such as stemness, invasion, and chemoresistance [12]. We have also reported that the regulation of breast cancer stemness may be controlled by PEBP, particularly through the upregulation of anti-inflammatory miR-145 and the downregulation of oncomiR-210 expression in vitro [12]. Additionally, we found that PEBP increases the expression of miR-200b in metastatic B16F10 skin cancer cells, an miRNA that is commonly downregulated in the melanoma cell line [13]. Consistent with our previous findings, we found that the PCA-based mixture significantly upregulated the expression of the tumor suppressor miR-145 in tumor samples of mice.

miR-145 is downregulated in various types of tumors, including breast tumors [38]. It plays an important role in the anti-tumorigenic functions of the FOXO1 transcription factor pathway, which regulates cellular proliferation, differentiation, apoptosis, and metastasis [39]. miR-145 suppresses metastasis in cancer by targeting various signaling pathways and suppressing multiple oncogenes. For instance, N-cadherin is a direct target of miR-145 [40], and its expression has been shown to be closely linked with invasion and metastasis in breast cancer tumors [41]. Moreover, the suppression of N-cadherin by miR-145 has been found to reduce cell invasion in breast cancer [42]. Additionally, the inhibition of ZEB2 by miR-145 allows for the expression of E-cadherin, which is known to inhibit cell migration in breast cancer [43,44]. We have previously shown that PEBP significantly inhibits the metastasis of 4T1 cells to the lungs in Balb/c mice [6]. The highly metastasizing 4T1 cell line typically forms metastasis in multiple organs such as the lungs, liver, and brain [45]. Consistent with our previous finding, we demonstrated that a polyphenolic mixture can inhibit the invasion of 4T1 cell to the lungs in a mouse model of breast cancer.

Furthermore, we studied the pathways related to miR-145, including FOXO1 and N-ras, in the breast cancer cell lines. FOXO1 downregulation occurs in various types of cancers [46]. For example, Dong et al. (2017) demonstrated that FOXO1 can inhibit cell motility, invasion in vitro, lung metastasis in vivo, and suppressed epithelial-to-mesenchymal transition (EMT) induced by ZEB2 [47]. Additionally, Li et al. (2019) reported that FOXO1 reduced tumor stemness and EMT signals in nasopharyngeal carcinoma by inducing miR-200b [48]. PI3K/AKT-mediated suppression of FOXO3A leads to expansion of the CSC population and promotes their self-renewal and mammospheres formation abilities [49]. Our previous results revealed the effectiveness of PEBP in inhibiting CSC formation and suppressing cellular motility and invasiveness by upregulating miR-200b and downregulating ZEB1 in skin cancer cell lines [13]. Moreover, we demonstrated the role of PEBP in inhibiting breast cancer stemness by upregulating FOXO1 and downstream miR-145 in breast cancer cell lines [12]. Similarly, in this study, we observed a significant increase in FOXO1 expression in cancer cell lines exposed to different doses of our polyphenolic mixture.

Ras protein upregulation might be associated with tumorigenesis, invasion, and metastasis [50]. Oncogenic N-ras elevation correlates with poor clinical outcomes and poor breast cancer-specific survival. Evidence shows overexpression of N-ras in the triple-negative subtype of breast cancer as the most aggressive breast cancer subtype [51]. Epigenetic modifications participate in N-ras expression and activity in breast cancer. For example, a study found that miR-145 exhibited antitumor activity by inhibiting tumor angiogenesis, cell invasion, and tumor growth through post-transcriptional modification of N-ras and growth factors [38]. In our research, we found an increase in N-ras levels in MDA-MB-231 cells exposed to the higher concentration of polyphenolic compounds, despite the higher level of miR-145 observed in the tumors. This contradicts our previous finding, where PEBP reduced N-ras levels in the same cell lines [12]. PEBP is a highly complex product, and this discrepancy may be due to the presence of components in PEBP that are not present in our mixture. Further research is necessary to understand and optimize the composition of our mixture.

In conclusion, our findings show the chemoprevention potential of a PCA-based polyphenolic mixture works, at least partly, by decreasing the number of tumor-initiating cells and preventing metastasis through the upregulation of miR-145. Our data might suggest this polyphenolic mixture could act as a potent chemo-preventive agent. Finally, nutritional approaches enriched with bioactive polyphenol compounds may be a viable strategy for preventing cancer. 

## 4. Materials and Methods

### 4.1. Preparation of Blueberry Juices

Fresh and untreated lowbush blueberries (*Vaccinium angustifolium* Ait.) were purchased from Cherryfield Foods Inc. (Cherryfield, ME, USA). Following blending the fruit (100 g) in a Braun Type 4259 food processor, the mixture was centrifuged at 500× *g* for 10 min to remove skin and other insoluble particles and extract fruit juice. The resulting juice was sterilized using 0.22 µm filters (Millipore, Etobicoke, Ontario, Canada). *Rouxiella badensis* subsp *acadiensis* SV-53 formally known as *Serratia vaccinii* bacterium was cultured as previously described [8]. The juice was inoculated with a saturated culture of the bacterium corresponding to 2% of the total juice volume. After four days of fermentation, the transformed juice was sterilized by 0.22 um filtration. The total phenolic content was then measured by the Folin-Ciocalteau method using gallic acid as standard and hence expressed as μM Gallic Acid Equivalent (GAE). Blueberry and biotransformed blueberry juice have been partially characterized elsewhere [8,52].

### 4.2. Metabolite Selection

An extensive literature survey was carried out to select the compounds previously reported in *Vaccinium* species using online databases KEGG, NIST, Scifinder, and Chemspider. This resulted in a wide variety of chemical classes, including anthocyanins, flavonoids, phenolic acids, phenolic glycosides, tocopherols, tocotrienols, terpenoids, and procyanidins. The abundance of metabolites was measured in fermented blueberry juice and compared with controls (non-fermented). Previously unreported compounds in *Vaccinium* were identified as the metabolites that were discriminant and changed in response or produced due to fermentation. 

### 4.3. Sample Preparation

Standards (>95% purity) of blueberry compounds, purchased from Sigma (Oakville, ON, Canada) and Extrasynthese Inc. (Lyon, France), were prepared at three dilutions that bracket the metabolite response in the samples. Blueberry juice was diluted 10-fold by Milli-Q water in a 5 mL glass tube, sonicated for 5 min, incubated at room temperature for 5 min, pipetted into a 96-well plate for analysis, and 5 µL of juices were injected. 

### 4.4. Ultra-Performance Liquid Chromatography-Quadrupole Time-of-Flight Mass Spectrometry (UPLC-MS-QTOF) Analysis

Analyses were undertaken on an Acquity UPLC coupled with a XevoG2 QTOF system (Waters Inc., Milford, MT, USA). UPLC analyses were performed on a Waters Acquity System. Separations were performed on a BEH C18 1.7 µm, 2.1 ×100 mm column (part #186002352; serial #02113226415705, LANSET# General Purpose 2.1 × 100 BEH) connected with a VanGuard pre-column 2.1 × 5 mm with the following characteristics: mobile phase A, water + 0.1% formic acid, B-acetonitrile + 0.1% formic acid (Fisher Optima LC-MS), flow rate 0.8 mL/min (back pressure at starting conditions = 10,000 PSI), column temperature, 65 °C, sample temperature 4 °C. Mobile phase B composition was 0–1 min 2% isocratic, 1–4 min linear gradient 2–20%, 4–9 min 20–40%, 9–11 min 40–60%, 11–14 min 60–100%, 14–18 min 100% isocratic. A 5 µL PLUNO injection was performed through a 10 uL loop followed by strong wash 200 µL (50% acetonitrile + 50% water) and weak wash 600 µL (10% acetonitrile+90% water).

Optimized Q-TOF analysis conditions were as follows: MassLynx software, MSe ESI+; and ESI- modes, lock mass Leucine Enkephalin ^12^C 556.2615, source temperature 150 °C; desolvation temperature 500°C; cone gas (N2) flow 50 L/hr; desolvation gas (N2) flow 1200 L/hr; MSe conditions, mass range 100-1500 Daltons; Low energy F1 conditions (CE, 6V, F2 CER 10-30V, cone voltage 20V, Scan time 1 sec); Instrument calibration; 50–1000 Da sodium formate. 

### 4.5. Cell Culture

Murine 4T1 and human MDA-MB-231 cell lines were obtained from the American Type Cell Collection (ATCC; Chicago, IL, USA). Cells were grown in RPMI-1640 media containing FBS (10%, *v*/*v*) (Sigma-Aldrich, Oakville, ON, Canada), penicillin/streptomycin (0.05 mg/mL) (Fisher Scientific, Toronto, ON, Canada) at 37 °C in a humidified atmosphere with 5% CO_2_. 4T1 and MDA-MB-231 were treated with 1- and 2-mM GAE of a polyphenolic mixture (PCA mix) containing PCA, gallic acid, and catechin for 24 h. Then, cells were collected to conduct relevant experiments. 

### 4.6. In-Vivo Breast Cancer Model

In this experiment, mice were maintained and treated in accordance with the guidelines of the Canadian Council on Animal Care. The protocol (HSe-3178) was approved by the Animal Care Committee of the University of Ottawa.

A total of 24 female Balb/c mice (Charles River, Montreal, QC, Canada), aged 6–8 weeks and weighing 18–20 g, were divided into two experimental groups (12 mice per group), including 1—control, receiving drinking water, and 2—receiving a polyphenolic mixture (a protocatechuic acid-based mixture) dissolved in drinking water. The mixture consisted of PCA (70 mg/kg BW), gallic acid (35 mg/kg Bw), and catechin (1.5 mg/kg Bw). After 2 weeks of feeding, animals were subcutaneously injected with 4T1 cells (1400 cells /0.2 mL/mouse) into the abdominal mammary gland fat pad, and nutritional intervention continued for three weeks. Then, mice were monitored for 3 weeks for tumor growth and health. At the end of the experiment, mice were euthanized, and the tumors and lungs were collected for further testing. All the tissues were digested using collagenase, and the resulting cells were cultured either to form mammospheres or in a 6-thioguanine enriched medium to detect the lung metastasis. 

### 4.7. Mammospheres Formation

4T1 and MDA-MB-231 cell lines were cultured in RPMI-1640 media until they reached 70% confluency. Then, adherent cells were detached using trypsin, and single cells were counted using Countess (Invitrogen, Burlington, ON, Canada). The cells were then seeded in ultra-low attachment 96-well plates (Corning, Saint-Laurent, QC, Canada) at 103 cells/0.2 mL/well, in the presence/absence of the PCA mixture (1- or 2-mM GAE), in DMEM-F12 Thermo Fisher Scientific. ON, Canada), supplemented with 10 ng/mL EGF (Millipore Sigma, Oakville, ON, Canada), 20 ng/mL bFGF (Millipore Sigma, Oakville, ON, Canada), 5 µg/mL insulin, 1 mM sodium pyruvate (Millipore Sigma, Oakville, ON, Canada), 0.5 µg/mL hydrocortisone (Millipore Sigma, Oakville, ON, Canada), and penicillin/streptomycin (0.05 mg/mL). Formed spheroids were counted after 2 to 3 days by light microscopy.

For tumor tissues, approximately 0.05g of each tumor was minced and dissociated in RPMI-1640 media containing 300 U/mL collagenase (Millipore Sigma, Oakville, ON, Canada), and 100 U/mL hyaluronidase (Millipore Sigma, Oakville, ON, Canada) at 37 °C for 2 h. Cells were sieved sequentially through 100 µm and 40 µm cell strainers (Fisher Scientific, Toronto, ON, Canada) to obtain a single cell suspension. Then, the single cells were plated at the same condition as above. Cells grown in these conditions formed non-adherent spherical clusters of cells or mammospheres, which were counted after 4–7 days.

### 4.8. Lung Metastasis

Lung metastasis was assayed as previously described [6]. Briefly, lungs were dissociated in RPMI-1640 media containing 300 U/mL collagenase, at 37 °C for 15 min. After filtration through a 40 µm cell strainer, the cells were gathered and resuspended in RPMI-1640 medium supplemented with 10% FBS, penicillin/streptomycin (0.05 mg/mL), and 60 μM 6-thioguanine (Millipore Sigma, Oakville, ON, Canada). The cells were plated in 10-cm sterile culture dishes and incubated at 37 °C and 5% CO_2_ for 14 days. Then, after fixation in methanol, cells were stained with 0.03% methylene blue solution. All blue colonies were counted, one colony representing one clonogenic metastatic cell in the lungs [45].

### 4.9. MicroRNAs Expression

The expression of miRNAs in breast tumors collected from mice was measured using qRT-PCR. Tumor samples RNA was extracted using a miRNeasy kit (Qiagen, Toronto, ON, Canada). Samples underwent a reverse transcription reaction to produce cDNA using individual probes. The cDNA was synthesized by Moloney Murine Leukemia Virus (MMLV) reverse transcriptase (Invitrogen, Burlington, ON, Canada). The expressions of miR-145 (TaqMan^®^ MicroRNA Assays 002278, Applied Biosystems, Burlington, ON, Canada) and miR-210 (TaqMan^®^ MicroRNA Assays 462444_mat, Applied Biosystems, Burlington, ON, Canada) were measured by RT-qPCR using Taqman primers (Applied Biosystems, Burlington, ON, Canada) and a FastStart Taq Polymerase (Roche, Mississauga, ON, Canada) in a CFX96 machine (Bio-Rad, Mississauga, ON, Canada). Gene expression was normalized to U6 small non-coding RNA as reference gene (Applied Biosystems, Burlington, ON, Canada).

### 4.10. Western Blot Analysis

4T1 and MDA-MB-231 cell lines were treated with different doses of the above-mentioned polyphenolic mixture (protocatechuic acid-based mixture) for 24 h. Cell lysates were extracted and run on a 4–12% acrylamide gel (Life Technologies, Burlington, ON, Canada), transferred to a PVDF membrane, probed with anti-FOXO1 (1:1000), anti-N-ras (1:1000), and anti-β-tubulin primary antibodies (1:1000) (Cell Signaling Tech. Inc., Danvers, MA, USA) and incubated at 4 °C overnight. The next day, blots were incubated with horseradish peroxidase-conjugated secondary antibodies (1:10,000) (Jackson Immuno Research Laboratories, West Grove, PA, USA) at room temperature for 1 h. Then, bands were visualized by chemiluminescence technique using ECL substrate (Bio-Rad, Mississauga, ON, Canada). Bands were quantified by the Bio-Rad Quantity One software using β-tubulin as the loading control.

### 4.11. Statistical Analysis

GraphPad Prism 5.0 software (GraphPad Software Inc., San Diego, CA, USA) was used to perform statistical analysis. Independent *t*-test was conducted to compare the means of two experimental groups, and one-way analysis of variance (ANOVA) followed by Dunnett’s post hoc test was performed to compare the means of more than two groups. Statistical significance was set at *p* ≤ 0.05. Data are reported as mean ± SEM. 

## Figures and Tables

**Table 1:** Characteristics of the identified metabolites in the fermented blueberry Juice. * denote parent peak.

Metabolite	Elemental Compound	Accurate Mass	Rt (min)	Ions Detected
Gallic acid	C7H6O5	170.0215	1.30	171.0293 (1+)/171.0295; 169.0137 (1−)/169.0131
Delphinidin-3-*O*-galactoside	C21H21O12+	465.1033	2.30	465.1033 (1+)/465.1025; 463.08752 (1−)/463.0873
Protocatechuic acid	C7H6O4	154.0266	2.31	155.0344 (1+)/155.0350; 153.0188 (1−)/153.0185
Idaein	C21H21O11+	449.1084	2.50	449.1084 (+)/449.1081; 447.0930 (2−)/447.0928
Catechol	C6H6O2	110.0368	2.58	111.0446 (1+)/112.9555, 130.9658; 109.0290 (1−)/109.0289
Cyanidin-3-*O*-glucoside	C21H21O11+	449.1084	2.58	449.1084(+)/449.1070; 447.0912 (2−)/447.0911
Salidroside	C14H20O7	300.1209	2.66	301.1287 (+)/323.1111 [M+Na]+1; 299.1131 (1−)/299.1138, 398.0308
Pyrocatechol-*O*-β-D-glucopyranoside	C12H16O7	272.2600	2.69	273.0974 (1+)/295.0791 [M+Na]+, 326.0083; 271.0818 (1−)/271.0818
Primulin	C23H25O12+	493.1346	2.83	493.1346(+)/493.1347, 331.0813
Oxycoccicyanin	C22H23O11+	463.1240	2.84	464.1319 (+)/463.1225; 462.1162 (2−)/461.1078
(+)-Catechin	C15H14O6	290.0790	2.88	291.0869 (1+)/291.0868,311.0532 * [M+Na]+1; 289.0712 (1−)/289.0717
p-hydroxybenzoic acid	C7H6O3	138.0317	2.88	139.0395 (1+)/139.0395; 137.0239 (1−)/137.0237
Oenin	C23H25O12+	493.1346	2.89	493.1346 (+)/493.1351; 491.1183(2−)
Procyanidin B2	C30H26O12	578.1424	3.08	579.1503 (+)/579.1509; 577.1346 (2−)/577.1359
Chlorogenic acid	C16H18O9	354.0951	3.12	355.1029(1+)/355.1008, 378.0843 [M+Na]+1; 353.0873 (1−)/353.0856,
(−)-Epicatechin	C15H14O6	290.0790	3.17	291.0869(1+)/291.0866; 289.0712 (1−)/298.0710
Myricetin-3-*O*-galactoside	C21H20O13	480.3800	3.47	481.0982(1+)/481.0973,503.0801 *; 479.0826 (1−)/479.0826
Myricetin-3-*O*-glucoside	C21H20O13	480.3800	3.51	481.0982 (1+)/481.0980; 479.0826 (1−)/479.0826
Quercetin-3-D-galactoside	C21H20O12	464.0955	3.80	465.1033(1+)/465.1035,303.0509 *, 487.0849; 463.0877 (1−)/463.0871
Quercetin-3-glucoside	C21H20O12	464.0955	3.83	465.1033 (1+)/465.1012, 303.0496 *; 463.0877 (1−)/463.0877
Quercetin-3-*O*-rhamnoside	C21H20O11	448.1006	4.13	449.1084 (1+)/449.1086, 471.0895, 303.0502 *; 447.0927 (1)/447.0923
Quercetin	C15H10O7	302.0427	4.91	303.0505 (1+)/303.0498; 301.0348 (1−)/301.0354

## Data Availability

The data presented in this study are available on request from the corresponding author.
